# Fear of being infected with COVID-19 virus among the medical social workers and its relationship to their future orientation

**DOI:** 10.3389/fpsyg.2022.985202

**Published:** 2022-09-06

**Authors:** Yaser Snoubar, Oǧuzhan Zengin

**Affiliations:** ^1^Social Work Program, Department of Social Sciences, College of Arts and Sciences, Qatar University, Doha, Qatar; ^2^Department of Social Work, Faculty of Economics and Administrative Sciences, Karabük University, Karabük, Turkey

**Keywords:** COVID-19, medical team, fear, future orientation, medical social worker

## Abstract

COVID-19 has been studied extensively for its direct effects on healthcare workers. Despite this, very little is known about the effect of COVID-19 fear on future orientation. Studying medical social workers’ fear of being infected with COVID-19 and their future orientation was the primary method used to examine this relationship. 204 Turkish medical social workers on the pandemic’s front lines were included in the total sample. Social workers were found to be extremely concerned about contracting COVID-19. Although only gender is a significant predictor of the fear of contracting COVID-19 infection, the study also found that female social workers have a higher fear of infection than males. Also, no correlation exists between social workers’ vaccination status and their fear of contracting COVID-19. There was a weak negative correlation between social workers’ fear of contracting COVID-19 and their future orientation, but in general, social workers had a positive future orientation. Medical social workers and front-line health care providers can use these findings to develop effective and culturally appropriate intervention programs to reduce COVID-19 concerns and foster a forward-looking outlook.

## Background

In health care settings, social work has a critical role to play. During the COVID-19 pandemic, social and psychological needs of patients and their families were of the utmost importance. Yet the pandemic and the virus’s spread have created numerous barriers to social justice, health care system incompetence, social exclusion, and racism for hospital social workers, despite their methods for providing support to patients. As a result of these challenges, social workers face a variety of situations in which they work with the health care team, including in the home (Ross H. et al., 2021). As a result, the COVID-19 pandemic had a significant impact on the clinical role of the social worker in supporting family members who had lost loved ones in the pandemic. In spite of this influence, social workers in the medical field were inspired to look for creative solutions through technology and innovation in clinical work (Fox et al., 2021; Snoubar, 2021). Since oncology patients are more susceptible to infection with COVID-19 due to their weakened immune systems, the use of a video phone to communicate with these patients has raised questions about whether or not direct patient contact is the best method for delivering the best care. The social worker had difficulty assessing body language and the inadequacy of feedback in this type of communication. Dissatisfaction with the job and negative thoughts about a future social work practice may result from this challenge. However, some useful alternatives have been developed that help patients communicate with their families (Di Ciero, 2021; Snoubar, 2021). In cases where these alternatives helped patients improve their emotional and social wellbeing by giving them access to safe technological means of communicating with their loved ones (Walter-McCabe, 2020), it was still difficult to help patients with cancer who were being diagnosed over the phone by an interpreter who had limited ability to read body language, recognize anxiety, communicate empathically, and correct misunderstandings as they occurred (Boparai et al., 2021). Many families, especially those disadvantaged or marginalized, lack access to technology and thus cannot benefit from these services as the rest of the population does. This may hinder the attainment of social justice in the health care field, despite the positive outlook on these innovations in the use of technology in social work practice remotely. This may put the social worker concerned about the future of social justice (Liburd et al., 2020). This crisis has led to new roles and mechanisms for medical social workers to create a supportive work environment and enhance cooperation among the various team members (Chen and Zhuang, 2020).

As this disease has affected their and their clients’ wellbeing, the COVID-19 pandemic has caused anxiety in social workers in various fields, particularly health care. As a result of the stress and sadness brought on by the pandemic, many social workers found it difficult to practice and adhere to normal standards, and they also suffered from a lack of motivation (Aaslund, 2021). The National Association of Social Workers (NASW) has provided numerous resources to assist social workers in addressing the fears and anxieties of the pandemic. Other resources available on the homepage include advocacy for COVID-19, legal resources for people in special populations and those with special health needs, social work safety, and the use of technology to support clients. Self-care during the Coronavirus Pandemic and ethics resources are also available (NASW, 2020). Additionally, the medical field’s response to the COVID-19 pandemic has been characterized by innovation and renewal by devising the most effective ways to mitigate the pandemic’s severity and enabling them to obtain informal support from family and friends.

In spite of the fact that medical social work is one of the oldest areas of social work science and practice, it did not show the desired progress in Turkey due to a lack of academic studies and professional personnel (Zengin, 2011). Therefore, conducting a wide range of studies on medical social workers in Turkey is critical to gathering data that can help improve their performance and increase their sense of wellbeing in the field (Ceylan et al., 2016; Gokler, 2021). However, this crisis has left social workers with many negative effects that require dealing with them for social workers to maintain their wellbeing and avoid diseases related to stress and mental health. It’s important to look at social workers’ negative feelings and fears about COVID-19 to determine what level of compatibility and stability is necessary for social workers to face the future positively. Medical social workers’ perspectives on the future, shaped by their experiences working on the front lines during the pandemic, are the focus of this study’s future orientation.

## The impact of COVID-19 on the medical social workers

Preliminary research shows that the pandemic has psychological and social effects that accelerate the spread of the epidemic and lead to high levels of depression and anxiety in the general population. Affected by COVID-19, health care workers are among the most vulnerable to mental health issues and require early psychosocial intervention (Ornell et al., 2020). Social workers, as well as other members of the health care team, have been negatively impacted by the pandemic. In addition to emotional exhaustion and depersonalization, which is considered a risk factor for mental health and their impact on the quality of their professional life, occupational burnout, and post-traumatic stress syndrome are among the most important of these negative effects that appear on the health care team during direct work on the front lines (Buselli et al., 2020; Carmassi et al., 2020; Johnson et al., 2020; Luceño-Moreno et al., 2020; Salehi et al., 2021). Social workers who work in emergency rooms may be more vulnerable to these effects, as they may experience anxiety and fear of contracting the disease (Shaukat et al., 2020). Medical social workers are an essential part of the health care delivery team because of the nature of their services and the roles they play (Herod and Lymbery, 2008). They aid those who are afflicted by long-term, life-threatening illnesses in obtaining the treatment they require and coping with the emotional and physical consequences of their condition for themselves and their loved ones (Alagaban, 2018). Working in a multidisciplinary team has led to the development of these roles, which have the primary responsibility of providing information about mental illnesses. It was the social workers’ job to connect patients and their families with medical professionals. The responsibilities of this role varied depending on the context in which they performed it as part of the multidisciplinary team (Gehlert, 2011). In times of epidemics and pandemics, social work can have a significant impact on the mental health of the public. The social worker helps to alleviate feelings of fear and anxiety that are linked to a person’s overall sense of wellbeing. There is a pressing need for social workers in this time of crisis to do things that will help people build or rebuild their capacities by creating the right social conditions (Barker, 1999). However, in the crisis of the COVID-19 pandemic, social workers faced the most difficult times in the history of social work by working in the front lines with the multidisciplinary health care team face to face with cases infected with the virus and in a stressful atmosphere (Redondo-Sama et al., 2020). Family support strategies, proactive meetings and contact between dying people and their loved ones are some of the psychosocial support interventions that can be used before and after death (Selman et al., 2020). In the course of treating those infected with the virus, social workers played a particularly delicate and crucial role. They bolstered the medical team’s support for patients and their families by implementing these interventions (Johns et al., 2020). Social workers are one of the most vulnerable to burnout and personal safety risks because of their position and the roles they play in the health care team (Queen and Harding, 2020). It’s difficult and stressful to deal with the effects of this pandemic on patients and coworkers, and doing so necessitates support and wellbeing maintenance through good self-care (Vo, 2021). Mental health needs to be prioritized and fatigue from working with patients must be eliminated if social workers are to continue fulfilling the responsibilities they have as members of the specialized team in their full potential (Hansel, 2020). During the COVID-19 pandemic, self-care by social workers is an essential tool for coping with stress and anxiety about the disease itself. It is critical that social workers learn and practice self-care practices in these trying times. People who work in healthcare will be under increasing pressure to manage their own wellbeing while also trying to help others, so it is essential that social workers develop strategies for managing their own wellbeing as the pandemic continues (Miller and Reddin Cassar, 2021). For social workers, this could have a significant impact on their mental health and future direction. Vaccination and the development of many vaccines may decrease fear of disease and increase a positive outlook toward the future.

## The study

This study aims to examine the relationship between fear of being infected with COVID-19 and the level of future orientation for the medical social workers and their relationship to some demographic and social variables. Following are the sub-goals of this research:

1. Recognize the degree of fear of being infected with COVID-19 in the social workers.
2. Recognize the degree of future orientation of the social workers.
3. Identify the relationship between the degree of fear of being infected with COVID-19 and the level of future orientation of the social workers.

In light of these objectives, this study seeks to answer the following questions:

1. What is the degree of fear of being infected with the COVID-19 among the social workers?
2. What is the degree of future orientation for the social workers?
3. Is there a statistically significant relationship between the degree of fear of being infected with COVID-19 among the social workers and each variable (age-gender—number of years of experience—vaccination status)?
4. Is there a statistically significant relationship between the level of future orientation of the social workers and each of the variables (age—gender—number of years of experience—vaccination status)?
5. Is there a statistically significant relationship between the degree of fear of being infected with COVID-19 and the level of future orientation of the social workers?

## Methodology

### Sample

This quantitative, cross-sectional, and descriptive study was designed to examine the fear of being infected with COVID-19 and their future orientation levels of medical social workers. For this purpose, the data of social workers in Turkey were collected using an electronic questionnaire due to the COVID-19 pandemic conditions. As of 2012, 600 social workers are working under the Ministry of Health in Turkey (Topuz and Öz, 2014). There is no data on the current number. Since the Ministry of Health employs an average of 100 new social workers every year, it is thought that approximately 1,600 social workers work in the Ministry of Health today. By accessing the online platforms used by social workers working in the state hospitals affiliated to the Ministry of Health, 204 social workers were reached with the improbable, purposive sampling method.

### Study materials

After reviewing studies that are relatively close to the subject of the study, the researchers found that because the subject of the current study was relatively recently studied, the previously developed measures or tests were incompatible with the subject. Additionally, the pre-made measure or test represents the task to be measured and other tasks, so it might not be relevant for the current study task. Also, we found that some pre-made tests whose standards are derived from a sample differ in nature from the sample being studied. Therefore, this study is based on using a scale designed by researchers after reviewing the research and studies conducted in the past 2 years on the negative effects of COVID-19 infection on the social workers. The research team also reviewed the scale to ensure its relevance to the cultural and social context of the sample under study. The scale consists of 17 phrases that are divided into two parts:

The first section, which consists of 8 statements, is concerned with measuring the degree of fear of being infected with COVID-19.

The second section, which consists of 9 phrases, is concerned with measuring the degree of future orientation.

In addition to the primary data (gender, age, marital status, number of years of experience, vaccination status).

### Procedure

Quantitative research methods were used to collect data from social workers working in the medical field in Turkey. Questions were developed to collect demographic data, and a 17-statement scale was used to collect data on fear of infected with COVID-19 and future orientation. In preparing the scale, many scales (Alipour et al., 2020; Chandu et al., 2020; Kumar et al., 2020; Nikčević and Spada, 2020; Silva et al., 2020) were viewed that we benefited from in designing the scale statements. The scale was written in Turkish; we sent the scale to two academics whose academic interests are social work practices in the field of health and then pre-test. The questionnaire was distributed to all social workers working in the medical field electronically through several channels and access their communication groups, emails, and phone calls. Informed consent is also placed at the beginning of the questionnaire, which indicates the confidentiality of information, how it is stored and used, and the social worker’s right to withdraw at will. The data collection process lasted from September 15, 2021, to October 15, 2021, and during this process, 204 social workers were reached.

### Statistical analysis

In the analysis phase of the data, firstly, missing data analysis was performed, and it was seen that there was no missing data. Descriptive statistics and histogram graphs were used to examine the fear of being infected with COVID-19 and the level of future orientation of healthcare workers. Multiple regression analysis was used to determine whether age, gender, and vaccination status significantly predicted fear of being infected with COVID-19 and future orientation. In the regression analysis, the predictors were included in the analysis simultaneously. Gender and vaccination status were included in the analysis as dummy variables. For the gender variable, the male category was determined as the reference category (female = 1, male = 0), while the non-vaccinated category (vaccinated = 1, unvaccinated = 0) was determined as the reference category for the vaccination status variable.

The assumptions of the multiple regression analysis were checked. As a result of the analysis, it was seen that there was no extreme value. Durbin-Watson values showed that the errors were independent in both regression models. When the standardized residuals histogram and normal P-P graph diagrams are examined, it is seen that the errors show a distribution similar to the normal distribution. Standardized predicted values and scatter plots of standardized residuals showed that the data provided homoscedasticity and linearity assumptions of variances. When the VIF (variance inflation factor) and tolerance values are examined, it is seen that there is no multi-collinearity problem between the variables.

### Research ethics

Ethical approval and written consent were obtained from the Research Ethics Committee of… University (2021/15.09). Informed consent was obtained from all participants in the study. Confidentiality was maintained by not requesting names or any other information identifying the social workers involved. The subjected were informed of their right to withdraw from the investigation at any time.

## Findings

**FIGURE 1:**
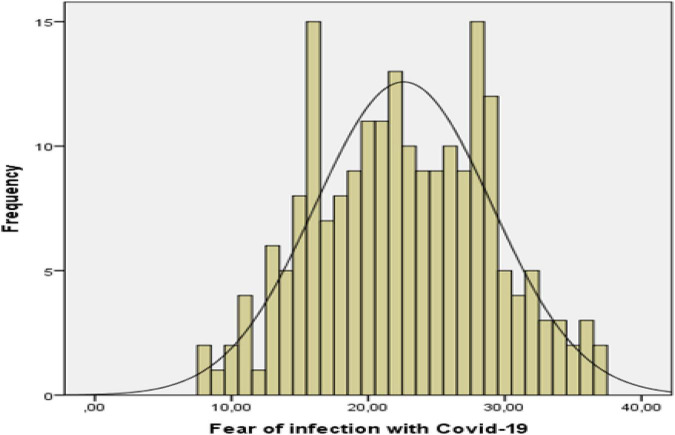
Histogram of fear of being infected with COVID-19 scores.

**FIGURE 2:**
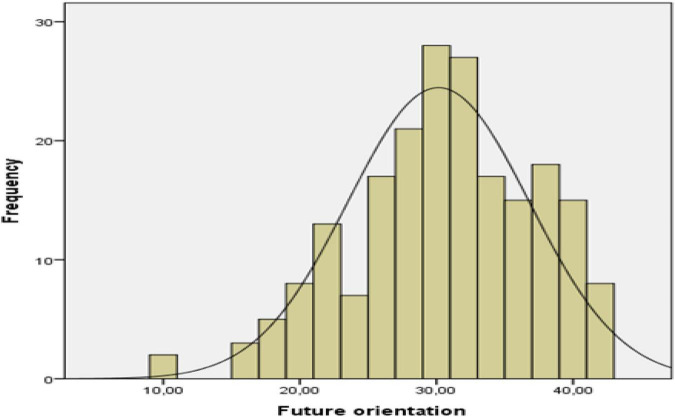
Histogram of future orientation scores.

Descriptive statistics were determined in order to examine the fear of being infected with COVID-19 and the level of future orientation of social workers working in the field of health. In addition, histogram charts showing the score distributions of social workers were used. The results of descriptive statistics are given in Table 1, and histogram graphics are given in Figures 1, 2.

**TABLE 1:** Descriptive statistics of fear of being infected with COVID-19 and future orientation variables.

	Fear of being infected with COVID-19	Future orientation
*N*	204	204
Average	22.593	30.152
Standard deviation	6.468	6.654
Median	22.00	30.00
Skewness	0.022	–0.434
Kurtosis	–0.627	–0.130
Min	8.00	10.00
Max	37.00	42.00
Range	29	32

As seen in Table 1, the skewness value of the distribution (0.022) for fear of being infected with COVID-19 is close to zero. The fact that the skewness value is close to zero indicates that the fear of being infected with COVID19 scores generally gather around the average. When the histogram graph in Figure 1 is examined, it is seen that the majority of the scores are around the mean. Accordingly, it can be inferred that social workers’ fear of being infected with COVID-19 is generally at a moderate level. The skewness value of the distribution of forward orientation scores (–0.434) and the histogram in Figure 2 shows that the distribution is slightly skewed to the left. The skewed distribution to the left indicates that the scores are relatively high. Accordingly, it can be deduced that the expectations of social workers for the future are generally positive.

Table 2 shows that the social workers who took part in the study had an average age of 30.82 years, but they had been in the field for an average of 6.92 years. A bachelor’s degree is held by nearly three-quarters of the participants, despite the fact that women make up slightly more than half (54.9 percent). Of the participants, only 28.9 percent had the COVID-19 virus, while 89.2 percent had the vaccine. Almost a quarter of the social workers (25.5%) said they didn’t want to work in health facilities because of the pandemic and instead wanted to work in another field.

**TABLE 2:** The social demographics of the sample.

	*N*	%
**Age**		
22–25	42	20,6
26–28	66	32,4
29–34	52	25,5
35 and above	44	21,5
**Gender**		
Female	114	55,9
Male	90	44,1
**Marital status**		
Single	106	52
Married	98	48
**Educational level**		
Bachelor’s degree	157	77
Postgraduate	47	23
**Being infected with COVID-19**		
Yes	59	28,9
No	145	71,1
**Getting a COVID-19 vaccine**		
Yes	182	89,2
No	7	3,4
Indecisive	15	7,4
**Field change request due to pandemic**		
Yes	52	25,5
No	152	74,5

Multiple regression analysis was performed to identify the variables that predicted social workers’ fear of contracting COVID-19. The standard (β) and non-standard (B) regression coefficients obtained as a result of the analysis are given in Table 3.

**TABLE 3:** Results of multiple regression model predicting fear of being infected with COVID-19.

The dependent variable	Predictor	B	SE	β	*t*	*P*
Fear of being infected with COVID-19	Constant	20.166**	2.465		8.183	<0.001
	Age	0.028	0.065	0.031	0.435	0.664
	Sex (Female = 1)	2.767*	0.925	0.213	2.992	0.003
	Vaccination status (Vaccinated = 1)	0.016	1.449	0.001	0.011	0.991

R = 0.208, R^2^ = 0.043.

F(3, 200) = 3.010, p = 0.031.

*p < 0.01, **p < 0.001.

As seen in Table 3, the created multiple regression model predicts the dependent variable significantly [*F*(3, 200) = 3.010, *p* = 0.031]. Variables in the model explain 4.3% of the variance in fear of being infected with COVID-19 (R^2 = .043). Of the variables in the model, only gender significantly predicts fear of being infected with COVID-19. Female social workers have a higher fear of being infected with COVID-19 than males (β = 0.213, *p* = 0.003). Age (β = 0.031, *p* = 0.664) and vaccination status (β = 0.001, *p* = 0.991) do not significantly predict fear of being infected with COVID-19. According to this finding, there is no relationship between age and vaccination status and fear of being infected with COVID-19.

Multiple regression analysis was performed to determine the variables that predicted the future orientation of social workers. The standard (β) and non-standard (B) regression coefficients obtained as a result of the analysis are given in Table 4.

**TABLE 4:** Results of multiple regression model predicting future orientation.

The dependent variable	Predictor	B	SE	β	*t*	*P*
Future orientation	Constant	30.496*	2.584		11.803	<0.001
	Age	0.032	0.068	0.035	0.476	0.634
	Sex (Female = 1)	–0.650	0.970	–0.049	–0.671	0.503
	Vaccination status (Vaccinated = 1)	–1.093	1.519	–0.051	–0.719	0.473

F(3, 200) = 0.422, p = 0.737.

R = 0.079, R^2^ = 0.006.

*p < 0.001.

As seen in Table 4; multiple regression model, in which age, gender and vaccination status variables were predictors, did not significantly predict the dependent variable [*F*(3, 200) = 0.422, *p* = 0.737]. Age (β = 0.035, *p* = 0.634), gender (β = There was no significant relationship between –0.049, *p* = 0.503) and vaccination status (β = –0.051, *p* = 0.473) with future orientation.

Pearson product-moment correlation coefficient was calculated to determine the relationship between fear of contracting COVID-19 and future orientation. As a result of the analysis, it was found that there was a weakly significant negative relationship between these two variables (*r* = –0.29, *p* < 0.001).

## Discussion

The COVID-19 pandemic has caused many burdens on people. Among these people, health personnel are at the forefront. Social workers working in the field of health are also included in this group. Increasing responsibilities and risks in both work and home life negatively affected social workers (Dubey et al., 2020; Pedrosa et al., 2020; Urooj et al., 2020). In a qualitative study conducted by Ross A. M. et al. (2021) with social workers working in hospitals, social workers had to cope with feelings of overwhelm and powerlessness due to the conditions and uncertainty they were in during the pandemic process. At the same time, safety concerns regarding the risk of exposure to COVID-19 were evident during this period. In our study, it can be said that social workers’ fear of being infected with COVID-19 is at a significant level. In the model established for the relationship of sociodemographic variables with the fear of being infected with COVID-19 in our study, only gender among the sociodemographic variables significantly predicts the fear of being infected with COVID-19. In this direction, the fear of being infected with COVID-19 among female social workers was found to be higher than that of males. This situation may be related to the excess of responsibilities related to home life from the perspective of women’s gender. This result agrees with Aughterson et al.’s (2021) study indicated the continuous and exacerbated anxiety of female health workers due to the fear of transmitting the virus to them and thus transmitting it to their families and loved ones. Also, it agrees with Abdelghani et al.’s (2020) study results, which indicates a higher level of fears of infection of COVID-19 among the health care team in general and an increase in these fears among females in particular. In addition, one study indicated that social workers fear that their older relatives and other family members may infect COVID-19 because of their direct work in the medical field (Senreich et al., 2021). This finding can also be linked to female fear of the stigma associated with COVID-19 in Eastern societies compared to Western societies (Wahed et al., 2020). According to our findings, there is no significant relationship between the vaccination status of social workers and the fear of being infected with COVID-19. This situation can be explained by the existence of social workers who do not believe in vaccines and diseases and therefore have less fear of being infected with COVID-19.

As stated in the study of Ross A. M. et al. (2021), social workers believe that there is a better future for the post-pandemic period. In our study, the expectations of social workers for the future are generally positive. However, no significant relationship was found between sociodemographic and vaccination status variables and future orientation. In addition to all these, as a result of the analysis carried out to determine the relationship between the fear of being infected with COVID-19 and the future orientation, it was found that there was a weakly significant negative relationship between these two variables. The study was conducted during the period when the effectiveness of vaccination against COVID-19 was proven, and cases of infection decreased globally, which may have impacted the future orientation.

### Strengths and limitations

To the authors’ knowledge, the current study is the first to investigate the relationship between fear of being infected with COVID-19 and future orientation in medical social workers. Besides, many limitations must be identified. This study was based on the quantitative approach, the mixed method may be more suitable for this type of study, but the conditions resulting from COVID-19, the precautionary measures, and the pressures faced by social workers working in the medical field due to the pandemic were taken into account when designing the methodology. However, we recommend that studies be conducted in a mixed manner to determine the concerns and concerns of social workers and their attitudes toward the future. Furthermore, the sample was identified with social workers working in the medical field, and this may not be generalized to mean social workers working in various fields during the pandemic.

### Conclusion and implications for social work and health

Since this study is one of the first studies examining the relationship between social workers’ fear of being infected with COVID-19 and the trend toward the future, it contributes to the social work literature in the medical field. The findings of this study provide indicators that alert social workers to the potential association between fear of contracting COVID-19 and future orientation to help develop psycho-spiritual assessments that are in line with the cultural context with a supportive focus for females based on their health, family, and community status. These results draw attention to the necessity of conducting research and developing policies focusing on the role of working with serious medical conditions in the results of the future orientation of female social workers.

Evidence indicates the spread of fear and mental health-related diseases among the medical team during the COVID-19 pandemic. Therefore, the medical care team must be aware of the relationship between fear of being infected with COVID-19 and future orientation. This may be beneficial for social work practice as social workers are in the process of preparing to deal with the mental health implications associated with the COVID-19 pandemic. For example, a recent study found that social workers could work with client emergencies despite a lack of resources and remain committed to providing services to their clients despite their concerns and situations related to their personal and family life (Senreich et al., 2021). However, the severe stresses associated with the pandemic and brought about by the nature of work in the medical field underscores the need for social workers for an integrated tool to alleviate the stresses associated with COVID-19 and anxiety about the disease itself. Self-care is one of the most important pillars that empower social workers and prepare them to support clients and the health care team (Miller and Reddin Cassar, 2021). All healthcare workers need psychological counseling and comprehensive mental health services because of their risk of developing PTSD and developing a range of negative consequences of COVID-19 (McFadden et al., 2021). Where social workers can contribute to providing support to the health care team by developing the policies of the institution, it is also assumed that social workers develop knowledge and practice skills related to the mental health needs of the health care team, such as trauma-informed care practices for group and individual trauma (Bender et al., 2021). Social worker intervention may be beneficial in supporting the wellbeing of health care workers, helping them manage emotional stress, and relieving fears (Aughterson et al., 2021). This is important because our current study found that social workers’ fear of being infected with COVID-19 has reached a significant level. Therefore, the social worker needs strategies to deal with these expected feelings when working on the front lines to fight against the disease. Also, female social workers may be more at risk of developing a mental disorder associated with working in the front lines in the medical field than males. Thus, social workers need to provide support to each other.

## Data availability statement

The original contributions presented in this study are included in the article/supplementary material, further inquiries can be directed to the corresponding author.

## Ethics statement

The studies involving human participants were reviewed and approved by the ethical approval and written consent were obtained from the Research Ethics Committee of Karabük University (2021/15.09). The patients/participants provided their written informed consent to participate in this study.

## Author contributions

YS and OZ contributed to the conception and design of the study and wrote sections of the manuscript. YS organized the database and wrote the first draft of the manuscript. OZ performed the statistical analysis. Both authors contributed to manuscript revision, read, and approved the submitted version.
